# Dual trajectories of post-traumatic growth and mental health in hysterectomy patients: a longitudinal study

**DOI:** 10.3389/fpubh.2026.1943852

**Published:** 2026-10-07

**Authors:** Yuqing Sun, Qinghua Guo, Yuanxiu Chen, Jianong Yu, Weizhi Fan, Jialin Jiang, Haixia He, Dingrong Qiu, Guanglian He

**Affiliations:** 1 Department of Gynecology, Ersha Island Hospital, The Second Affiliated Hospital of Guangzhou University of Chinese Medicine, Guangzhou, China; 2 Nursing Department, The Second Affiliated Hospital of Guangzhou University of Chinese Medicine, Guangzhou, China

**Keywords:** dual trajectories, hysterectomy, longitudinal Studies, mental health, post-traumatic growth

## Abstract

**Background:**

Research indicates that undergoing a hysterectomy increases a woman's risk of developing long-term mental health issues, such as anxiety and depression. At the same time, there is currently a lack of research exploring their mental health from the perspective of positive psychology, such as post-traumatic growth. This study aims to examine the types of developmental trajectories of post-traumatic growth and mental health among hysterectomy patients. It also employs a dual-trajectory model to explore the longitudinal associations between the two trajectories.

**Methods:**

The study enrolled hysterectomy patients at the gynecology department of a tertiary hospital in Guangdong Province between February and December 2024. A general demographic questionnaire, the post-traumatic growth inventory-Chinese version, and the 12-item general health questionnaire were administered at 1 week post-surgery, 1 month post-surgery, and 3 months post-surgery. A total of 220 patients were enrolled, and 190 ultimately completed the follow-up. The group-based trajectory model analyzed the dynamic trajectories of post-traumatic growth and psychological wellbeing among patients, as well as the relationship between these two trajectories. The study was reported using the STROBE checklist.

**Results:**

The post-traumatic growth trajectories of hysterectomy patients were categorized into four groups: “very low level rising group” (14.0%), “low level rising group” (35.4%), “medium level rising group” (35.1%), and “high level stable group” (15.5%). Mental health trajectories were categorized as “good mental health rising group” (16.8%), “mental health risk reduction group” (62.5%), and “psychological problem relief group” (20.7%). Among patients in the very low level rising PTG group, the probability of being classified into the psychological problem relief group was 49.5% (95% CI: 8.3%−64.3%). Patients in the good mental health rising group had a 42.6% (95% CI: 11.7%−74.5%) probability of being classified into the high level stable group. These results indicate that the two trajectories are highly correlated.

**Conclusion:**

This study demonstrates that the trajectories of post-traumatic growth and mental health among hysterectomy patients are heterogeneous. The dynamic interdependence between post-traumatic growth and mental health can significantly impact patients' quality of life and overall health.

## 1 Introduction

Hysterectomy is one of the primary and preferred treatment options for gynecological conditions (1). The number of surgeries has been increasing annually, with demand projected to grow further in the future. However, hysterectomy may damage the patient's pelvic floor support structures, affect ovarian function and certain nerve functions, and increase the risk of postoperative complications such as pelvic floor dysfunction, urinary dysfunction, and sexual dysfunction (2, 3). It may also lead to the early onset or exacerbation of perimenopausal symptoms (4), impacting patients' emotional regulation (5). Pressures from family and society can also impact patients' self-perception and role adjustment, altering their mental health status (6). Research indicates that women who undergo a hysterectomy face a higher risk of developing long-term mental health issues, such as anxiety and depression, compared to those who don't undergo the procedure (7).

Mental health is an integral part of overall wellbeing. Poor psychological status poses significant challenges to postoperative recovery and disease outcomes in patients, adversely affecting their quality of life (8). The China Women's Development Outline (2021–2030) states that efforts should be made to further safeguard and promote women's mental health literacy (9). Therefore, from both individual and societal perspectives, prioritizing the mental health of patients who have undergone a hysterectomy is of paramount importance. However, previous research has predominantly focused on the negative psychological aspects of hysterectomy patients, with a notable lack of studies exploring their psychological wellbeing from a positive psychology perspective.

Many researchers have found that individuals battling illness not only experience negative emotions but also undergo positive psychological changes known as “post-traumatic growth” (PTG). Post-traumatic growth can mitigate the impact of negative psychological distress on patients, alleviate their psychological suffering (46), and hold significant value in improving patients' quality of life (10–12). The theoretical model of post-traumatic growth emphasizes that post-traumatic growth is a variable that changes with the traumatic event and over time (13). Exploring the mechanisms underlying the occurrence and development of post-traumatic growth can provide deeper insights into how to effectively enhance the mental health of patients who have undergone hysterectomy. However, research on the positive psychological development process following hysterectomy remains relatively scarce. Previous studies on hysterectomy patients have only examined overall post-traumatic growth changes (14), overlooking the possibility that the overall group may contain distinct subgroups and that developmental trajectories may differ across these subgroups (15, 16). Furthermore, due to differences in study design and analytical methods, research findings remain controversial. Meanwhile, an individual's mental health can fluctuate over time. The trajectory of mental health is also influenced by individual differences (17–19). Describing the mental health trajectory of hysterectomy patients facilitates the implementation of personalized interventions for this population. Some studies have examined the trajectories of depressive symptoms specifically in patients who have undergone hysterectomy and have identified three distinct trajectories (20). However, there are still relatively few studies that focus on the specific trajectories of overall mental health in hysterectomy patients. Research findings indicate that post-traumatic growth is highly correlated with an individual's mental health status, with the two often influencing each other (21). Notably, the dynamic relationship between them remains underexplored. This is a critical issue that warrants in-depth investigation using longitudinal research designs.

The group-based trajectory model (GBTM) is commonly used to analyse trajectories in longitudinal data. GBTM can explicitly identify the developmental trajectories and trajectory characteristics of various subgroups within a population, making it suitable for psychometric data (22). The dual-trajectory model can explore the relationship between two longitudinal data variables. This method simultaneously accounts for the dynamics and heterogeneity between variables, enabling the investigation of how these time-varying variables interact over time (23).

In summary, this study aims to collect data on patients' mental health and post-traumatic growth across different stages through a longitudinal survey. Using group trajectory models, we will identify trajectory categories for changes in post-traumatic growth and mental health among hysterectomy patients, and explore the longitudinal associations between these two trajectories. Identifying the developmental trajectory and characteristics of psychological states in hysterectomy patients enables early identification and management of high-risk individuals. This provides a theoretical foundation for developing targeted psychological intervention programs and improving patients' mental health and quality of life.

The following are the hypotheses of this study: (1) Both post-traumatic growth and mental health among hysterectomy patients change over time, with distinct trajectory subgroups emerging; (2) We hypothesize that, across different subgroups, lower trajectories of post-traumatic growth are associated with lower trajectories of mental health, and vice versa.

## 2 Methods

### 2.1 Design and sample

This study employed a longitudinal research design and was reported using the STROBE checklist. This study enrolled patients hospitalized for total hysterectomy in the Department of Gynecology at a tertiary hospital in Guangdong Province from February to December 2024. Inclusion criteria: (a) undergoing total hysterectomy; (b) age 18 years or older; (c) alertness and ability to complete questionnaires independently or with assistance; (d) provision of informed consent. Exclusion criteria: (a) participation in psychological intervention studies within the past 6 months; (b) combined with severe physical illness; (c) severe psychological disorders, psychiatric conditions, or cognitive impairment; (d) the family experienced other major traumatic events (such as bereavement, natural disasters, etc.). Meanwhile, during the follow-up period, participants who: (a) withdrew from the study midway; (b) didn't proceed as planned, or refused follow-up, were removed.

This study employed G^*^Power 3.1 for sample size calculation (24). Based on the study design, assuming *f* = 0.25 (medium effect size), α = 0.05, (1–β) = 0.90, with 4 trajectory categories (a conservative planning parameter based on prior studies reporting 3–4 latent trajectory groups in similar populations; this assumption was used only for sample-size planning and did not pre-specify the number of trajectory groups in the GBTM), 3 repeated measures, and a repeated measures correlation coefficient of 0.5 (moderate correlation), ε = 0.5, the minimum required sample size was calculated to be 80 cases. Considering a minimum of 20–30 subjects per group and a 20% dropout rate, the sample size should be at least 143.

### 2.2 Measurements

#### 2.2.1 General information questionnaire

This questionnaire was developed independently by the researchers based on existing literature. It includes demographic information, such as age, marital status, childbearing status, educational level, occupation, household income, residence, and payment methods, as well as disease-related information, including degree of knowledge of the disease, surgical indication, surgical approach, other chronic diseases, time since diagnosis, treatment methods, and length of hospital stay. Disease-related data were extracted from electronic medical records and confirmed with the attending physician where necessary. Surgical indication was classified as benign conditions (e.g., uterine fibroids, adenomyosis) or malignant conditions (e.g., endometrial cancer, cervical cancer). Surgical approach was classified as laparoscopic surgery (including total laparoscopic hysterectomy) or non-laparoscopic surgery (including total abdominal hysterectomy and vaginal hysterectomy).

#### 2.2.2 Post-traumatic growth inventory-Chinese version (C-PTGI)

The scale was developed by Tedeschi and Calhoun (25) and translated and revised by Wang et al. (26). It comprises five dimensions and totals 20 items. Each item is scored from 0 to 5, with higher total scores indicating greater levels of post-traumatic growth. Notably, the revised scale demonstrated good reliability and validity, as reflected in Cronbach's α coefficients, which ranged from 0.611 to 0.874 across dimensions and the total score. As a result, it has been widely adopted (27). In this study, Cronbach's α was 0.937.

#### 2.2.3 12-item general health questionnaire (GHQ-12)

The scale consists of 12 items describing an individual's life state. Participants report their level of agreement using a 4-point Likert scale based on their experiences over the past 4 weeks. Six items are phrased positively and are reverse-scored. The remaining six items are negatively worded and scored positively. The total scale score ranges from 0 to 36, with higher scores indicating poorer mental health. The dimensionality of this scale remains controversial; previous research has proposed a three-dimensional structure (27). In the present study, the total GHQ-12 score was used as a single indicator of mental health in all analyses; no separate subscale scores were modeled. The Cronbach's α coefficient in this study was 0.830.

### 2.3 Data collection

Before the formal commencement of the survey, the research team will conduct systematic training for survey personnel to standardize the investigation process. Following approval from hospital and department heads, a pilot survey will be conducted in February 2024. Based on the pilot survey results, the questionnaire and survey procedures will be refined, and the average completion time of the questionnaire will be determined.

The first formal survey was conducted within 1 week post-surgery. Investigators thoroughly explained the purpose and process of the survey to participants and informed them about subsequent follow-up arrangements. They also assured patients that survey results would be kept strictly confidential and obtained their informed consent. After completion, questionnaires were collected and retrieved by investigators for review. Disease-related data were entered by researchers based on electronic medical records. Post-discharge surveys were conducted at 1 month and 3 months post-surgery through outpatient follow-ups, telephone follow-ups, or electronic questionnaires. Patients attending outpatient follow-ups completed questionnaires on-site. Those unable to complete questionnaires on-site selected telephone or online questionnaires based on personal preference. During telephone follow-ups, interviewers asked each scale item sequentially, confirming responses with patients before recording.

### 2.4 Ethics approval

This study has been approved by the Ethics Committee of Guangdong Provincial Hospital of Traditional Chinese Medicine (YE2024−029) and is conducted in accordance with the Declaration of Helsinki. The research adheres to the principles of voluntariness, confidentiality, fairness, and benefit without harm. All research data will be strictly confidential and used solely for this study. Participants may withdraw from the study at any time without providing a reason.

### 2.5 Statistical analysis

Data were entered by two people using EpiData 3.1 software. Participants with any missing C-PTGI or GHQ-12 data at any of the three time points were excluded (complete-case analysis). Missing proportions and patterns were documented, and baseline characteristics and baseline C-PTGI and GHQ-12 scores were compared between completers and non-completers using appropriate tests; no significant differences were found. Missingness was considered likely MCAR; no imputation was performed because complete-case analysis is unbiased under MCAR. Questionnaires with logical errors were cross-checked against the original forms using their corresponding identification numbers. Descriptive analysis was conducted on the general characteristics, C-PTGI, and GHQ-12 scores. Frequency (%) was used for categorical data, and (*M* ± SD) was used for normally distributed continuous data. Data from patients completing follow-up were compared with those from lost-to-follow-up patients. For normally distributed variables, repeated-measures analysis of variance was used to assess differences in scores across the three time points, followed by pairwise comparisons.

GBTM is a method for analyzing the developmental trajectories of longitudinal indicators. It identifies potential trajectory subgroups within a population based on objective indicators. This approach overcomes the limitations of classical trajectory analysis, which only explains individual variations in the overall average trajectory while overlooking the potential existence of trajectory subgroups within a group. In recent years, it has been widely applied in the medical field (28). The fitting process begins with the assumption of a single trajectory category, progressively increasing the number of trajectory categories. Because only three measurement time points were available, the highest identifiable polynomial order was quadratic. Each trajectory was initially fitted using a quadratic function, and non-significant higher-order terms (*P* ≥ 0.05, Wald test) were removed sequentially, with the linear term retained regardless of significance.

Model selection was based on the Bayesian Information Criterion (BIC), with the smallest absolute BIC value indicating better fit (29), and the following criteria: (1) Average posterior probability (Ave PP): reflects the degree of alignment between an individual and its trajectory subgroup. Higher values closer to 1 indicate better performance; the study sets 0.7 as the acceptable threshold for model validity. (2) Odds of correct classification (OCC): An OCC >5 indicates high classification accuracy for that model. (3) Specified entropy ≥0.7, with values closer to 1 indicating better model fit. (4) The sample size of each subgroup should constitute no <5% of the total population, determined by the overall sample size and the shape of the trajectory subgroups. The optimal number of trajectory classes was determined by comparing models with increasing numbers of groups. The final model was selected based on the above criteria, with clinical interpretability considered.

After deriving separate trajectory models for the two variables, the parameters from both models are input as initial values into a dual-trajectory model for fitting. The association between the two variables across different horizontal trajectory groups is expressed as joint probabilities. Specifically, this includes: (1) the conditional probability that members of each trajectory group for Indicator A become members of each trajectory group for Indicator B; (2) the conditional probability that members of each trajectory group for Indicator B become members of each trajectory group for Indicator A; (3) the probability that a specific member belongs to both a specific trajectory group for Indicator A and a specific trajectory group for Indicator B. For the dual-trajectory model, point estimates were derived from the original model fitting, and 95% confidence intervals for conditional and joint probabilities were obtained using a non-parametric bootstrap with 1,000 resamples, resampling at the individual level. Percentile-based confidence intervals were calculated. Implemented using Stata MP 17. Additionally, logistic regression analysis was employed to examine the predictors of post-traumatic growth and mental health developmental trajectories using SPSS 25.0.

## 3 Results

### 3.1 Sample characteristics at baseline

This study included 220 patients who met the criteria. During the first survey conducted within 1 week postoperatively, 8 patients voluntarily withdrew from the study, and 4 questionnaires were excluded for failing to meet the criteria, resulting in a total of 208 returned questionnaires; during the 1-month postoperative follow-up, 11 patients were lost to follow-up, including 7 who could not be contacted and 4 who withdrew from the study; at the 3-month postoperative follow-up, 7 patients were lost to follow-up, including 3 who withdrew from the study and 4 who could not be contacted. During follow-up, 26 patients were lost to follow-up, and 4 problematic questionnaires were excluded. Ultimately, 190 patients completed all follow-up visits and were included in the analysis. The overall missing rate was 13.6%. The main reasons for missing data were loss to follow-up, voluntary withdrawal, and invalid questionnaires. A complete-case analysis was adopted; participants with any missing C-PTGI or GHQ-12 data at any of the three time points were excluded.

There were no significant differences between patients who completed follow-up and those who were lost to follow-up in terms of sociodemographic characteristics, disease-related information, baseline C-PTGI scores, or GHQ-12 scores (*P* > 0.05) (Table 1). These findings suggest that the missingness mechanism was likely missing completely at random (MCAR) or approximately MCAR.

**Table 1:** Patients' general information [*n* = 190, *n* (%)].

Variable	Category	Completed follow-up (*n* = 190)	Lost to follow-up (*n* = 18)	χ^2^/*t*	*P*
Age	<40	5 (2.6)	1 (5.6)	4.528	0.107
	40–59	154 (81.1)	11 (61.1)		
	≥60	31 (16.3)	6 (33.3)		
Marital status	Married	159 (83.7)	16 (88.9)	0.334	0.564
	Single/Divorce/Widowed	31 (16.3)	2 (11.1)		
Childbearing status	No	20 (10.5)	2 (11.1)	0.006	0.939
	Yes	170 (89.5)	16 (88.9)		
Education level	Elementary school and below	28 (14.7)	4 (22.2)	3.230	0.357
	Junior high school	47 (24.7)	5 (27.8)		
	High school or technical secondary school	58 (30.5)	2 (11.1)		
	College degree or above	57 (30.0)	7 (38.9)		
Occupation status	Employed	55 (28.9)	3 (16.7)	2.608	0.456
	Retired	61 (32.1)	6 (33.3)		
	Unemployed or jobless	29 (15.3)	2 (11.1)		
	Other	45 (23.7)	7 (38.9)		
Average monthly household income	<3,000 yuan	32 (16.8)	3 (16.7)	0.007	0.996
	3,000–5,000 yuan	72 (37.9)	7 (38.9)		
	>5,000 yuan	86 (45.3)	8 (44.4)		
Residence	City	156 (82.1)	17 (94.4)	1.789	0.181
	Township or Rural	34 (17.9)	1 (5.6)		
Payment methods	At own expense	5 (2.6)	2 (11.1)	3.674	0.299
	Rural cooperative medical care	13 (6.8)	1 (5.6)		
	Resident medical insurance	61 (32.1)	5 (27.8)		
	Employee medical insurance	111 (58.4)	10 (55.6)		
Degree of knowledge of the disease	Basic understanding	144 (75.8)	13 (72.2)	0.113	0.737
	Partial understanding	46 (24.2)	5 (27.8)		
Surgical indication	Benign	149 (78.4)	11 (61.1)	2.775	0.096
	Malignant	41 (21.6)	7 (38.9)		
Surgical approach	Laparoscopic surgery	170 (89.5)	14 (77.8)	2.204	0.138
	Open surgery	20 (10.5)	4 (22.2)		
Other chronic diseases	No	144 (75.8)	13 (72.2)	0.113	0.737
	Yes	46 (24.2)	5 (27.8)		
Time since diagnosis	<1 year	79 (41.6)	10 (55.6)	1.371	0.504
	1–5 years	61 (32.1)	4 (22.2)		
	>5years	50 (26.3)	4 (22.2)		
Treatment methods	Surgery	165 (86.8)	13 (72.2)	2.847	0.092
	Surgery + Adjuvant therapy	25 (13.2)	5 (27.8)		
Length of hospital stay	≤ 7 days	51 (26.8)	3 (16.7)	3.395	0.159
	8–14 days	109 (57.4)	9 (50.0)		
	>14 days	30 (15.8)	6 (33.3)		
C-PTGI		55.19 ± 18.07	47.89 ± 15.06	−1.661	0.098
GHQ-12		10.38 ± 4.38	12.17 ± 6.35	1.169	0.257

The mean age of patients included in this study was 51.34 ± 7.911 years. The majority of patients were married (83.7%). 89.5% of patients had children. Educational level was as follows: 14.7% had primary school education or below, 24.7% had junior high school education, 30.5% had senior high school or technical secondary education, and 30% had college education or above. Employee medical insurance was the most common payment method (58.4%). Most patients underwent hysterectomy for benign conditions (78.4%). Regarding surgical approach, 170 patients (89.5%) underwent laparoscopic surgery, and 20 patients (10.5%) underwent non-laparoscopic surgery. See Table 1 for details. The post-traumatic growth scores for hysterectomy patients during the T1, T2, and T3 periods were (55.19 ± 18.07) points, (58.32 ± 13.90) points, and (64.91 ± 13.53) points, respectively; and their mental health scores were (10.38 ± 4.38), (7.44 ± 4.40), and (4.75 ± 4.62), respectively. Repeated-measures analysis of variance revealed significant differences in patients' scores for post-traumatic growth (*F* = 87.521, *P* < 0.001) and mental health (*F* = 155.927, *P* < 0.001) across the three time points. A *post hoc* comparison showed that the relationship among C-PTGI scores at the three time points was T3 > T2 > T1, while the relationship among GHQ-12 scores was T1 > T2 > T3.

### 3.2 Post-traumatic growth and mental health trajectories among hysterectomy patients

First, fix the degree of the polynomial to determine the optimal number of groups; second, fix the number of groups and select the polynomial degree that provides the best fit (28). The number of trajectory groups was determined by comparing models with 1–5 groups; the 4-group model had the smallest absolute BIC value (BIC = −2,200.33) among all group-number models. Then, we set the number of groups to 4, with each group initially fitting intercept, linear, and quadratic terms; non-significant quadratic terms were removed sequentially. The optimal polynomial degrees for each group of PTG trajectories were determined to be 2, 1, 1, and 0, respectively. The proportions for the four groups were 14.0%, 35.4%, 35.1%, and 15.5%, respectively, all exceeding 5%. The Ek was 0.816, >0.7. The Avepp for the four groups ranged from 0.87 to 0.94, while the OCC ranged from 12.097 to 79.339, both meeting ideal standards. This indicates that the model classification results are reliable. The fitting parameters for the post-traumatic growth trajectories are shown in Table 2.

**Table 2:** Fit statistics of the multigroup trajectories for post-traumatic growth.

Model	Avepp (%)	AIC	BIC	ΔBIC	Percentage of each group (%)	Ek
4_2 2 2 2	0.92/0.88/0.89/0.93	−2,174.35	−2,200.33	−2,158.35	14.4/36.0/34.2/15.4	0.821
4_2 1 1 1	0.90/0.89/0.88/0.93	−2,174.46	−2,195.56	−2,161.46	14.0/35.5/35.0/15.5	0.816
4_2 1 1 0	0.90/0.89/0.87/0.94	−2,173.68	−2,193.16	−2,161.68	14.0/35.4/35.1/15.5	0.816

AIC, akaike information criterion; BIC, Bayesian information criterion; Ave PP, average posterior probability; Ek, relative entropy.

The number of mental health trajectory groups was determined by comparing models with 1 to 4 groups; the 3-group model had the smallest absolute BIC value (BIC = −1,575.22) among all group-number models. Then, we set the number of groups to 3, with each group initially fitting intercept, linear, and quadratic terms; non-significant quadratic terms were removed sequentially. After multiple iterations of fitting, with non-significant quadratic terms removed sequentially, the optimal polynomial orders for each group's trajectory were determined to be 1, 2, and 1, respectively. The proportions of the three trajectories were 16.8%, 62.5%, and 20.7%, all exceeding 5%. The Ek was 0.768. The Avepp ranges for the three groups were 0. 80–0.92, and the OCC ranges were 6.953–34.835, indicating reliable model classification. The fitting parameters for the mental health trajectories are shown in Table 3. Table 4 presents the parameter estimates for the PTG and MH trajectory models, showing that all parameters were statistically significant.

**Table 3:** Fit statistics of the multigroup trajectories for mental health.

Model	Avepp (%)	AIC	BIC	ΔBIC	Percentage of each group (%)	Ek
3_222	0.80/0.92/0.90	−1,555.74	−1,575.22	−1,543.74	16.8/62.5/20.7	0.767
3_121	0.80/0.92/0.90	−1,553.77	−1,570.00	−1,543.77	16.8/62.5/20.7	0.768

AIC, akaike information criterion; BIC, Bayesian information criterion; Ave PP, average posterior probability; Ek, relative entropy.

**Table 4:** Parameter estimation of trajectory models for post-traumatic growth and mental health.

Class	Parameter	PTG [*Beta* (*SE*)]	PTG [*Wald* (*P*)]	MH [*Beta* (*SE*)]	MH [*Wald* (*P*)]
1	Intercept	26.947 (2.174)	12.397 (<0.001)	5.359 (0.678)	7.907 (<0.001)
	Linear	12.667 (3.428)	3.695 (<0.001)	−2.446 (0.410)	−5.968 (<0.001)
	Quadratic	−2.158 (1.064)	−2.028 (0.043)		
2	Intercept	48.036 (1.812)	26.511 (<0.001)	10.392 (0.398)	26.097 (<0.001)
	Linear	3.849 (0.506)	7.600 (<0.001)	−4.414 (0.648)	−6.814 (<0.001)
	Quadratic			0.722 (0.199)	3.632 (<0.001)
3	Intercept	61.641 (1.702)	36.221 (<0.001)	14.281 (0.548)	26.051 (<0.001)
	Linear	2.825 (0.544)	5.198 (<0.001)	−1.012 (0.282)	−3.589 (<0.001)
4	Intercept	81.023 (1.084)	74.712 (<0.001)		

PTG: Class 1 = very low level rising group; Class 2 = low level rising group; Class 3 = medium level rising group; Class 4 = high level stable group. MH: Class 1 = good mental health rising group; Class 2 = mental health risk reduction group; Class 3 = psychological problem relief group. PTG, post-traumatic growth; MH, mental health.

The trajectories of post-traumatic growth among hysterectomy patients are grouped as shown in Figure 1. Trajectories are named based on initial scores and change patterns as follows: class 1 scores start at extremely low levels and continuously increase, termed the “very low level rising group” (14.0%). Class 2 initial scores are below average and show an upward trend over time, named the “low level rising group” (35.4%). Class 3 exhibits initial scores at moderate levels that steadily increase over time, termed the “medium level rising group” (35.1%). Class 4 features initial scores at high levels that remain consistently stable, thus designated the “high level stable group” (15.5%).

**Figure 1:**
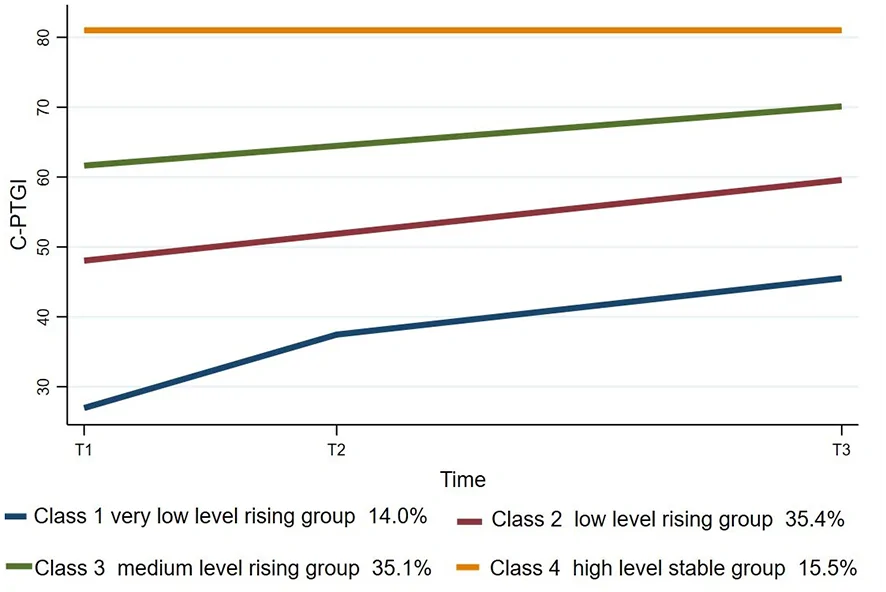
Trajectories of post-traumatic growth.

Figure 2 illustrates the trajectory patterns of mental health. Based on initial scores and trends, the groups are classified as follows: Class 1 has a low initial score that continues to decline, indicating that patients initially had a favorable mental state and that their mental health continued to improve over time. This group is designated as the “good mental health rising group” (16.8%). Class 2 has an initial score >10, indicating that patients are at risk of developing mental health issues postoperatively. Subsequently, the GHQ-12 score continued to decline, indicating that the risk of developing psychological problems gradually decreased. Therefore, this group is named the “mental health risk reduction group” (62.5%). The initial score of Class 3 exceeded 14 points, indicating that patients exhibit severe negative psychological states and hold a negative attitude toward postoperative recovery. Furthermore, their subsequent score decline is relatively small. Therefore, this group is named the “psychological problem relief group” (20.7%).

**Figure 2:**
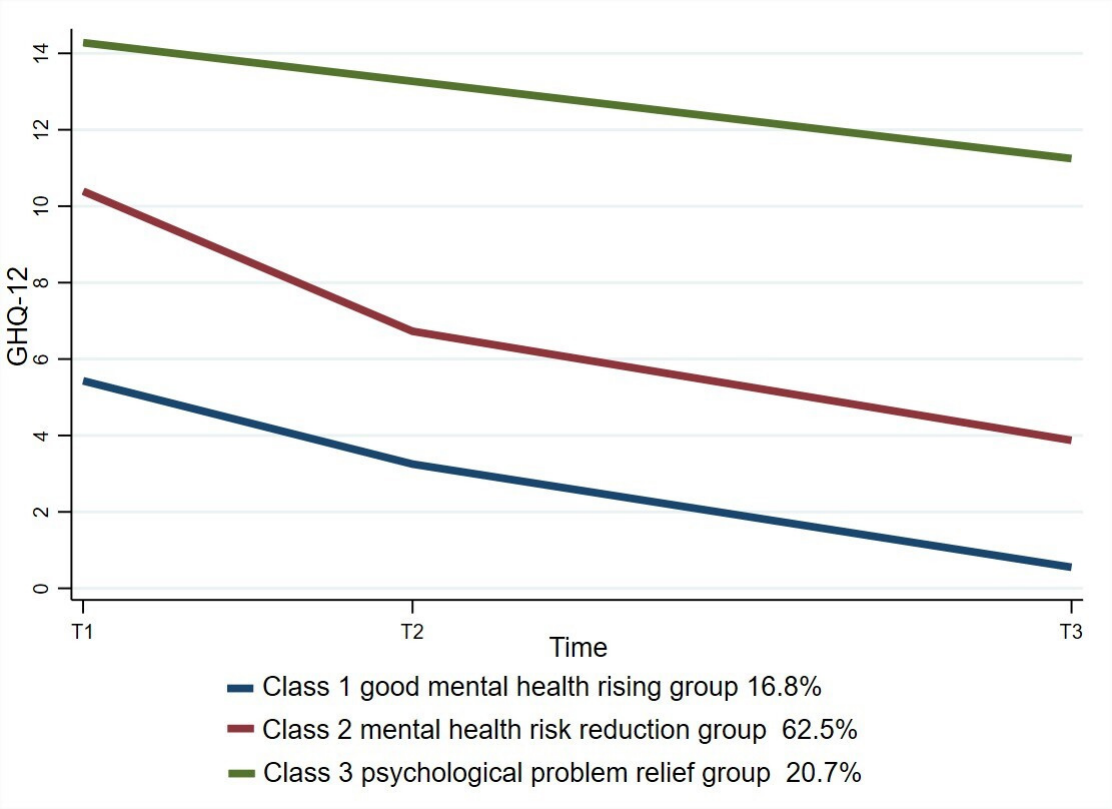
Trajectories of mental health.

### 3.3 Factors influencing the trajectory of post-traumatic growth

A univariate analysis was performed on the baseline demographic data. The results showed statistically significant differences (*P* < 0.05) in C-PTGI scores among patients in different groups with respect to age, educational level, average monthly household income, disease knowledge, other chronic diseases, and time since diagnosis (Supplementary Table S1).

We conducted an unordered multi-class logistic regression analysis, using the variables listed in Supplementary Table S2 as independent variables and trajectory categories as the dependent variable. The analysis shows that the time since diagnosis, average monthly household income, disease knowledge, other chronic diseases, and age are each significantly associated with different post-traumatic growth trajectories among hysterectomy patients.

When using the “high level stable group” as a reference, patients with an average monthly household income of <3,000 yuan are more likely to fall into the “very low level rising group”. Patients with an average monthly household income of 3,000–5,000 yuan were more likely to be classified into the “very low level rising group” or the “low level rising group”. In contrast, patients with a disease duration of no more than 5 years, a basic understanding of their condition, no chronic illnesses, and aged 40–59 were more likely to exhibit post-traumatic growth consistent with the “high level stable group”, as shown in Supplementary Table S3.

### 3.4 Factors influencing the trajectory of mental health

Univariate analysis revealed statistically significant differences (*P* < 0.05) among the different mental health trajectories in terms of occupation, average monthly household income, payment methods, surgical indication, surgical approach, degree of knowledge of the disease, treatment methods, and length of hospital stay (Supplementary Table S4). Logistic regression was performed using these variables as independent variables and mental health trajectories as the dependent variable. The values assigned to the independent variables are shown in Supplementary Table S5.

The results indicate that surgical indication, degree of knowledge of the disease, and treatment methods are predictive factors for different trajectories of mental health. Using the “psychological problem relief group” as the reference, diagnosis of benign condition and a basic understanding of the disease were found to be predictive factors for the “mental health risk reduction group”. A benign diagnosis and surgical treatment were predictive factors for the “good mental health rising group” (Supplementary Table S6).

### 3.5 Relationships between post-traumatic growth and mental health trajectories

These analyses describe the association between membership in the post-traumatic growth trajectory groups and membership in the mental health trajectory groups. They do not imply that individuals moved from one trajectory group to another over time. The probability of mental health trajectory membership conditional on post-traumatic growth trajectory membership is shown in Figure 3. Among patients in the very low level rising PTG group, the probability of being classified into the psychological problem relief group was 49.5% (95% CI: 8.3%−64.3%). In the low level rising group, 63.3% (95% CI: 40.2%−78.1%) of patients were classified into the mental health risk reduction group. The probability of patients in the medium level rising group being classified into the mental health risk reduction group was 77.7% (95% CI: 48.8%−86.3%). Among patients in the high level stable group, the probability of being classified into the psychological problem relief group was 4.0% (95% CI: 0.0%−18.0%).

**Figure 3:**
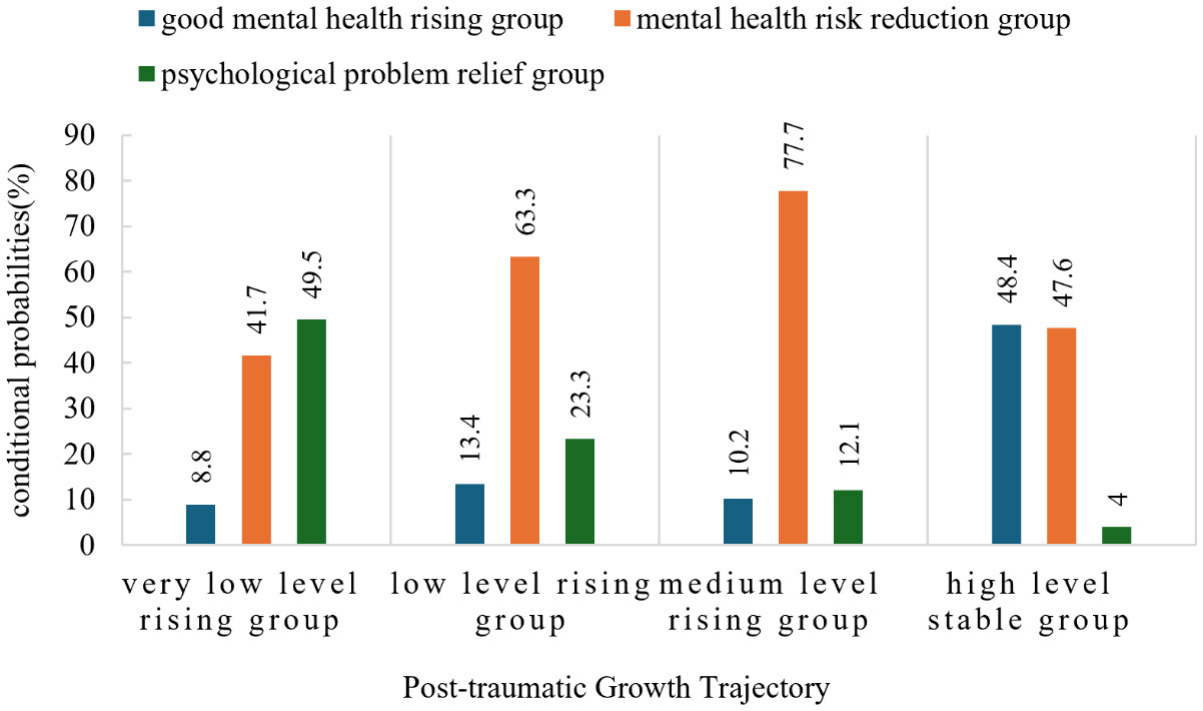
Probability of mental health trajectory groups under post-traumatic growth trajectory group conditions.

The probability of post-traumatic growth trajectory membership conditional on mental health trajectory membership is shown in Figure 4. Patients in the good mental health rising group had a 42.6% (95% CI: 11.7%−74.5%) probability of being classified into the high level stable group. Among the mental health risk reduction group, 42.7% (95% CI: 9.1%−53.2%) of patients were classified into the medium level rising group. Patients in the psychological problem relief group had a 3.0% (95% CI: 0.0%−16.7%) probability of being classified into the high level stable group. The joint probability of post-traumatic growth and mental health trajectory membership is shown in Figure 5. The most common joint classification was the medium level rising group combined with the mental health risk reduction group (26.7%, 95% CI: 12.6%−39.0%). The high level stable group combined with the mental health risk reduction group accounted for 7.0% (95% CI: 1.6%−14.2%). The very low level rising group combined with the good mental health rising group accounted for 1.3% (95% CI: 0.0%−4.2%). The high level stable group combined with the good mental health rising group accounted for 7.1% (95% CI: 1.1%−11.1%). The complete set of conditional and joint probabilities with 95% confidence intervals is provided in Supplementary Table S7.

**Figure 4:**
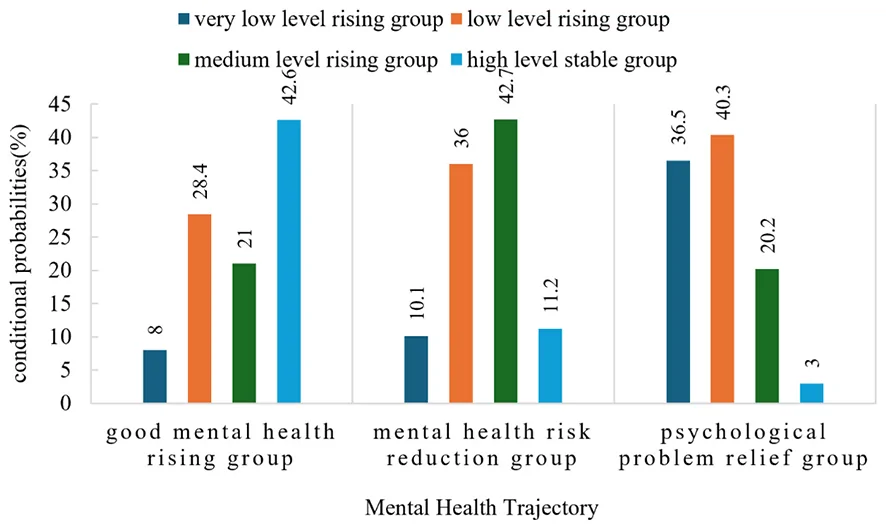
Probability of post-traumatic growth trajectory groups under mental health trajectory group conditions.

**Figure 5:**
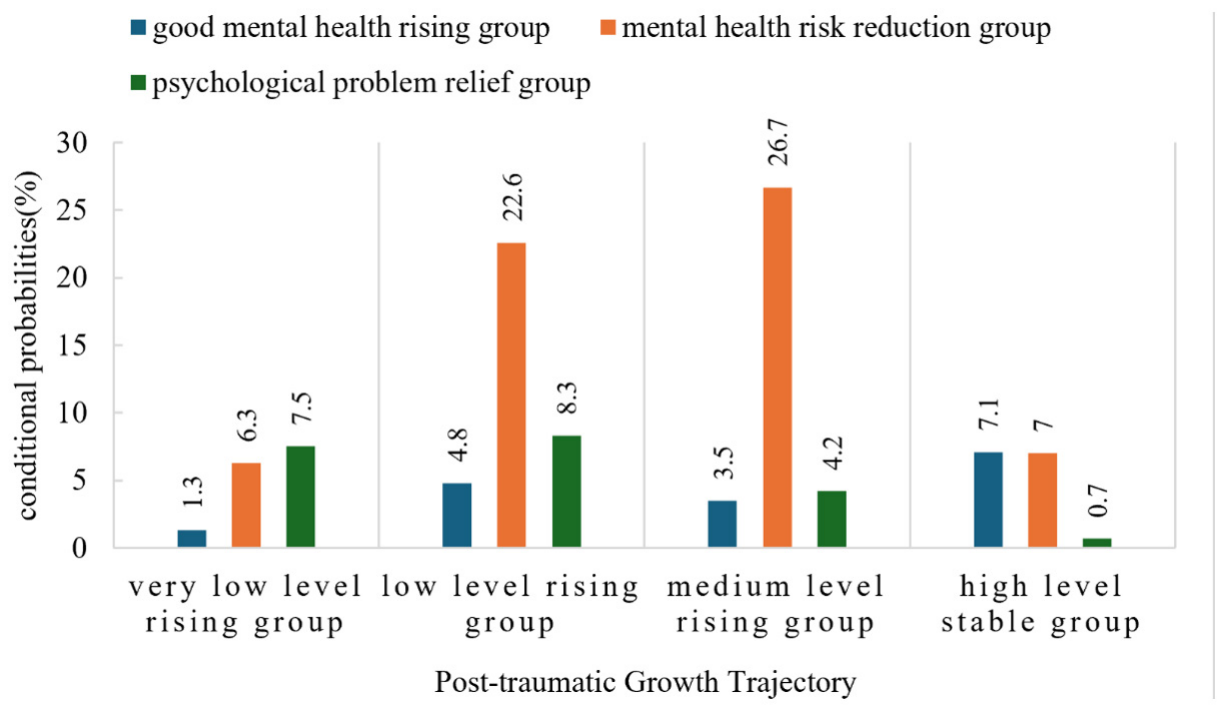
Joint probability of post-traumatic growth and mental health trajectory groups.

## 4 Discussion

This study examined the dynamic trajectories of post-traumatic growth and mental health among hysterectomy patients and identified factors associated with each trajectory. It also found a longitudinal association between the trajectories of post-traumatic growth and mental health.

### 4.1 Analysis of post-traumatic growth trajectories and influencing factors

The study findings revealed four distinct trajectories of post-traumatic growth among hysterectomy patients, showing both similarities and differences compared to previous research (15, 30, 31). These differences may stem from variations in the study populations and the timing of data collection. This also underscores the reliability of the present study's findings and demonstrates the heterogeneity in the development of post-traumatic growth among hysterectomy patients (15). The study further analyzed the factors influencing the trajectory of post-traumatic growth and found that this trajectory was influenced by the time since diagnosis, average monthly household income, disease knowledge, other chronic diseases, and age. These findings are consistent with those of previous studies (31, 32).

Previous studies suggest that patients with higher incomes may face a lighter financial burden, be able to recover in better environments, and experience less psychological stress (33). They may be more likely to share positive experiences following their illness, which may be associated with post-traumatic growth (34). Therefore, for patients with lower incomes, healthcare providers should do their best to help them choose cost-effective treatment options and teach them how to care for themselves after discharge, thereby reducing unnecessary expenses. For patients facing particularly severe financial hardship, hospitals can assist them in applying for social assistance. Compared to older patients, younger patients demonstrate a greater willingness to embrace new approaches. They are more proactive in making personal changes and show more significant progress. Therefore, when caring for older patients, healthcare providers should promptly identify and address their needs, provide appropriate education, and build their confidence. They should also ensure regular follow-ups and continuity of care after discharge. Furthermore, the more comprehensive a patient's understanding of their condition, the better they can objectively assess the severity of the situation. This may help alleviate patients' fears and may be associated with their personal growth (35). This suggests that healthcare professionals should assist hysterectomy patients in developing a positive understanding of their condition and encourage peer-to-peer communication and the sharing of experiences among patients. This will help foster a positive mindset among patients. This study also found that most patients with a longer duration of illness underwent surgery for benign conditions. These patients typically received conservative treatment initially, and the decision to undergo surgery was often driven by a diminished need for the uterus. Consequently, patients didn't experience significant emotional distress following the procedure. The combination of chronic conditions may be associated with a weakened immune system, causing their physical condition to deteriorate further and making them more prone to negative emotions. This may also affect the development of treatment plans, increase the patient's burden, and be associated with lower PTG. Therefore, healthcare providers should pay closer attention to patients with chronic conditions, provide adequate psychological support, and adjust care approaches based on the patient's physical condition.

### 4.2 Analysis of mental health trajectories and influencing factors

The trajectories of mental health in hysterectomy patients within the first 3 months post-surgery include three distinct types. Consistent with the findings of previous studies (17, 36), this demonstrates the heterogeneity of mental health trajectories among patients.

Surgical indication, disease understanding, and treatment methods were included in the logistic regression model, reflecting their unique significance for patients' mental health. These factors reflect the severity of the patient's condition. The poorer the self-reported physical health and the more severe the symptom burden, the worse the patient's psychological outcomes (37). A study by Ou (9) also showed that hysterectomy patients with a malignant diagnosis had higher anxiety and depression scores than those with a benign diagnosis who underwent surgery. Malignant diseases can trigger fear and other negative emotions in patients. Patients may need to undergo a series of follow-up treatments, which can place a significant physical and psychological burden on them. At the same time, patients may worry that, despite incurring substantial costs, they will still not achieve a favorable prognosis, which is not conducive to the development of a positive mindset. For patients with malignant diagnoses and complex treatment processes, healthcare providers should exercise greater patience, increase communication with patients, and instill confidence in their ability to return to normal life. Furthermore, research has shown that the more knowledge patients have about their condition, the less psychological stress they experience (38). The extent of this knowledge depends, to a certain degree, on the health education provided by healthcare professionals. Therefore, for patients with limited understanding of their condition, healthcare professionals should provide personalized disease education in addition to basic mental health education to alleviate their distress.

### 4.3 Relationships between post-traumatic growth and mental health trajectories

This study uses a dual-trajectory approach to explore the multidimensional relationships between the different trajectories of the two variables (39). Before interpreting these associations, it is important to note that the first assessment was conducted within 1 week post-surgery, a period of acute physical recovery. The initial trajectory levels of both post-traumatic growth and mental health should therefore be interpreted as early postoperative states rather than stable pre-surgery baselines. These levels may be influenced by acute pain, fatigue, hospitalization, and surgical stress, but this timing is unlikely to substantially alter the overall pattern of associations between the two trajectories. Future studies could include a preoperative baseline assessment to better distinguish acute surgical effects from longer-term psychological trajectories in hysterectomy patients. The results indicate that post-traumatic growth trajectory group membership was associated with mental health trajectory group membership among hysterectomy patients. For example, patients in the high level stable PTG group had a relatively high probability of being classified into the good mental health rising group, whereas patients in the very low level rising PTG group had a relatively high probability of being classified into the psychological problem relief group. These associations are directly supported by the present data. However, the directionality and causality of these associations cannot be determined from this observational study.

Previous literature has suggested that, as hysterectomy patients navigate their illness and surgery, post-traumatic growth may manifest as positive changes in self-perception and emotions. Patients' perspectives on interpersonal relationships and life may be reshaped, potentially prompting them to modify unhealthy lifestyle habits and enhancing their adaptive capacity. These changes may be associated with improved mental health and a favorable prognosis (40). Based on these previous findings, we hypothesize that patients with high levels of post-traumatic growth may engage in active, purposeful, and reflective processing when faced with a crisis, form positive cognitive evaluations, and adopt positive coping strategies. Furthermore, social support and positive psychological capital have been proposed as resources for developing positive post-traumatic cognition (41). Patients with good mental health may typically have sufficient confidence to overcome adversity and may be able to improve themselves through active learning. They may be able to employ effective strategies to cope with challenges, which may in turn be associated with personal growth. Therefore, they may be more likely to achieve positive post-traumatic growth (42). These hypothesized mechanisms require further investigation.

Changes in post-traumatic growth and mental health are associated with patients' quality of life and overall health. Therefore, healthcare providers should tailor interventions to the specific needs of different patient groups. For patients in poor condition, active psychological intervention and ongoing monitoring are essential, along with thorough post-discharge follow-up. Research has shown that psychological care approaches such as cognitive behavioral therapy and mindfulness-based stress reduction can clinically promote post-traumatic growth (43). These methods can help patients adjust their thinking and guide them toward facing their experiences with acceptance and understanding, thereby enhancing their psychological resilience (44). Accordingly, healthcare professionals can use proactive psychological care interventions to encourage patients to engage in appropriate cognitive restructuring and self-management, thereby further improving their overall health.

### 4.4 Strengths

First, by examining the psychological changes experienced by hysterectomy patients from the perspective of positive psychology, this research helps provide comprehensive psychological support for these patients. Second, this is the first study to examine the changes in post-traumatic growth and mental health among hysterectomy patients from a common longitudinal perspective. The study accounted for trajectory heterogeneity and established a dual-trajectory model. The findings provide new insights for the scientific and effective management of mental health in hysterectomy patients.

### 4.5 Limitations

Several limitations should be acknowledged. First, this single-center study relied on self-report data using general-purpose scales, which limits generalizability and may introduce bias; larger multicenter studies with more specific instruments are needed. Second, the follow-up period was limited to 3 months with only three assessments and no external validation, which limits inferences about long-term trends, causality, and the predictive validity of the trajectory models; future studies should extend follow-up, incorporate external validation, and use methods such as structural equation modeling. Third, complete-case analysis may reduce statistical power and introduce bias if missingness was not random; maximum likelihood estimation or multiple imputation should be considered. Fourth, small subgroup sizes produced wide 95% confidence intervals for some conditional and joint probabilities and precluded stratified analyses by surgical indication. In addition, ovarian status was not recorded (45), and social support, coping strategies, and cognitive appraisal were not measured; therefore, the mechanisms proposed to explain the association between the two trajectories are inferred from previous literature and should be validated in larger studies.

## 5 Conclusion

This study used GBTM to model the trajectories of post-traumatic growth and mental health in hysterectomy patients and to examine the factors associated with each trajectory and the association between the two trajectory memberships. The findings indicate that post-traumatic growth trajectory group membership was associated with mental health trajectory group membership. Specifically, patients in the higher PTG trajectory groups were more likely to be classified into the more favorable mental health trajectory groups, and vice versa. However, this association is descriptive and does not establish causality or directionality. This study provides a basis for understanding the longitudinal patterns of post-traumatic growth and mental health among hysterectomy patients, identifying high-risk groups prone to psychological issues at an early stage, and informing targeted psychological intervention plans. Future research with longer follow-up and external validation is needed to determine the predictive value of these trajectory models.

## Data Availability

The original contributions presented in the study are included in the article/Supplementary material, further inquiries can be directed to the corresponding authors.
