# Supplementary material for: Dual trajectories of post-traumatic growth and mental health in hysterectomy patients: a longitudinal study
Source: Front Public Health. 2026 Oct 7;14:1943852. doi: 10.3389/fpubh.2026.1943852 (PMC13693529; doi:10.3389/fpubh.2026.1943852)
Supplement: Supplementary file 1 [file Data_Sheet_1.docx]

Supplementary Material

Table S1. Univariate analysis of general characteristics in the post-traumatic growth trajectory of hysterectomy patients

| Variable | | C1（*n*=28） | C2（*n*=64） | C3（*n*=68） | C4（*n*=30） | *χ*^2^ | *P* |
| --- | --- | --- | --- | --- | --- | --- | --- |
| Age | <40 | 0（0.0） | 1（1.6） | 1（1.5） | 3（10.0） | 16.932 | 0.003 |
|  | 40-59 | 17（60.7） | 52（81.3） | 61（89.7） | 24（80.0） |  |  |
|  | ≥60 | 11（39.3） | 11（17.2） | 6（8.8） | 3（10.0） |  |  |
| Marital status | Married | 24（85.7） | 49（76.6） | 58（85.3） | 28（93.3） | 4.344 | 0.221 |
|  | Single/Divorce/Widowed | 4（14.3） | 15（23.4） | 10（14.7） | 2（6.7） |  |  |
| Childbearing status | No | 2（7.1） | 8（12.5） | 7（10.3） | 3（10.0） | 0.553 | 0.959 |
|  | Yes | 26（92.9） | 56（87.5） | 61（89.7） | 27（90.0） |  |  |
| Education level | Elementary school and below | 7（25.0） | 13（20.3） | 6（8.8） | 2（6.7） | 21.467 | 0.011 |
|  | junior high school | 7（25.0） | 19（29.7） | 15（22.1） | 6（20.0） |  |  |
|  | High school or technical secondary school | 10（35.7） | 18（28.1） | 25（36.8） | 5（16.7） |  |  |
|  | College degree or above | 4（14.3） | 14（21.9） | 22（32.4） | 17（56.7） |  |  |
| Occupation status | Employed | 3（10.7） | 16（25.0） | 23（33.8） | 13（43.3） | 12.193 | 0.222 |
|  | Retired | 11（39.3） | 19（29.7） | 25（36.8） | 6（20.0） |  |  |
|  | Unemployed or jobless | 5（17.9） | 11（17.2） | 9（13.2） | 4（13.3） |  |  |
|  | Other | 9（32.1） | 18（28.1） | 11（16.2） | 7（23.3） |  |  |
| Average monthly household income | <3000 yuan | 12（42.9） | 9（14.1） | 9（13.2） | 2（6.7） | 21.155 | 0.001 |
|  | 3000-5000 yuan | 8（28.6） | 31（48.4） | 26（38.2） | 7（23.3） |  |  |
|  | >5000 yuan | 8（28.6） | 24（37.5） | 33（48.5） | 21（70.0） |  |  |
| Residence | City | 22（78.6） | 52（81.3） | 55（80.9） | 27（90.0） | 1.612 | 0.657 |
|  | Township or Rural | 6（21.4） | 12（18.8） | 13（19.1） | 3（10.0） |  |  |
| Payment methods | At own expense | 2（7.1） | 1（1.6） | 1（1.5） | 1（3.3） | 7.819 | 0.515 |
|  | Rural cooperative medical care | 3（10.7） | 4（6.3） | 4（5.9） | 2（6.7） |  |  |
|  | Resident medical insurance | 10（35.7） | 25（39.1） | 19（27.9） | 2（23.3） |  |  |
|  | Employee medical insurance | 13（46.4） | 34（53.1） | 44（64.7） | 20（66.7） |  |  |
| Degree of knowledge of the disease | Basic understanding | 12（42.9） | 46（71.9） | 57（83.8） | 29（96.7） | 26.602 | <0.001 |
|  | Partial understanding | 16（57.1） | 18（28.1） | 11（16.2） | 1（3.3） |  |  |
| Surgical indication | Benign | 22（78.6） | 50（78.1） | 55（80.9） | 22（73.3） | 0.706 | 0.872 |
|  | Malignant | 6（21.4） | 14（21.9） | 13（19.1） | 8（26.7） |  |  |
| Surgical approach | Laparoscopic surgery | 24（85.7） | 56（87.5） | 62（91.2） | 28（93.3） | 1.398 | 0.727 |
|  | Open surgery | 4（14.3） | 8（12.5） | 6（8.8） | 2（6.7） |  |  |
| Other chronic diseases | No | 10（35.7） | 48（75.0） | 59（86.8） | 27（90.0） | 32.295 | <0.001 |
|  | Yes | 18（64.3） | 16（25.0） | 9（13.2） | 3（10.0） |  |  |
| Time since diagnosis | <1 year | 5（17.9） | 21（32.8） | 34（50.0） | 19（63.3） | 38.105 | <0.001 |
|  | 1-5 years | 4（14.3） | 27（42.2） | 23（33.8） | 7（23.3） |  |  |
|  | >5years | 19（67.9） | 16（25.0） | 11（16.2） | 4（13.3） |  |  |
| Treatment methods | surgery | 23（82.1） | 54（84.4） | 60（88.2） | 28（93.3） | 2.094 | 0.552 |
|  | Surgery + Adjuvant Therapy | 5（17.9） | 10（15.6） | 8（11.8） | 2（6.7） |  |  |
| Length of hospital stay | ≤7 days | 9（32.2） | 14（21.9） | 21（30.9） | 7（23.4） | 3.438 | 0.761 |
|  | 8-14 days | 13（46.4） | 40（62.5） | 37（54.4） | 19（63.3） |  |  |
|  | >14 days | 6（21.4） | 10（15.6） | 10（14.7） | 4（13.3） |  |  |

Note: C1=very low level rising group, C2=low level rising group, C3 = medium level rising group, C4=high level stable group.

Table S2. Assigning values to independent variables of post-traumatic growth trajectories

| Independent variable | Variable name | Assignment method |
| --- | --- | --- |
| Age | X_1_ | Using “≥60 years old” as the reference point |
|  |  | <40 years =1, 40-59 years =0 |
|  |  | <40 years =0, 40-59 years =1 |
| Education level | X_2_ | Using “college degree or above” as the reference point |
|  |  | Elementary school and below =1，junior high school =0，High school or technical secondary school =0 |
|  |  | Elementary school and below=0，junior high school =1，High school or technical secondary school =0 |
|  |  | Elementary school and below=0，junior high school =0，High school or technical secondary school=1 |
| Average monthly household income | X_3_ | Using “>5000 yuan” as the reference point |
|  |  | <3000 yuan =1，3000-5000 yuan =0 |
|  |  | <3000 yuan =0，3000-5000 yuan =1 |
| Degree of knowledge of the disease | X_4_ | Basic understanding=1，Partial understanding=2 |
| Other chronic diseases | X_5_ | No=1，Yes=2 |
| Time since diagnosis | X_6_ | <1year=1,1-5 years=0 |
|  |  | <1 year =0,1-5 years=1 |

Table S3. Logistic regression of post-traumatic growth trajectories

| Group | Variable | | *B* | *SE* | *Waldχ*^2^ | *OR* | 95%*CI* | | *P* |
| --- | --- | --- | --- | --- | --- | --- | --- | --- | --- |
|  |  |  |  |  |  |  | Lower | Upper |  |
| C1 VS C4 | Time since diagnosis | <1 year | -3.837 | 0.987 | 15.107 | 0.022 | 0.003 | 0.149 | <0.001 |
|  |  | 1-5 years | -2.588 | 1.048 | 6.095 | 0.075 | 0.010 | 0.587 | 0.014 |
|  |  | >5years | Ref |  |  |  |  |  |  |
|  | Average monthly household income | <3000 yuan | 3.916 | 1.185 | 10.920 | 50.215 | 4.921 | 512.424 | 0.001 |
|  |  | 3000-5000 yuan | 1.920 | 0.888 | 4.679 | 6.821 | 1.198 | 38.854 | 0.031 |
|  |  | >5000 yuan | Ref |  |  |  |  |  |  |
|  | Degree of knowledge of the disease | Basic understanding | -3.051 | 1.215 | 6.302 | 0.047 | 0.004 | 0.512 | 0.012 |
|  |  | Partial understanding | Ref |  |  |  |  |  |  |
|  | Other chronic diseases | No | -3.959 | 0.969 | 16.682 | 0.019 | 0.003 | 0.128 | <0.001 |
|  |  | Yes | Ref |  |  |  |  |  |  |
|  | Age | 40-59 | -2.020 | 0.943 | 4.590 | 0.133 | 0.021 | 0.842 | 0.032 |
|  |  | ≥60 | Ref |  |  |  |  |  |  |
| C2 VS C4 | Time since diagnosis | <1years | -1.787 | 0.713 | 6.284 | 0.167 | 0.041 | 0.677 | 0.022 |
|  |  | >5 years | Ref |  |  |  |  |  |  |
|  | Average monthly household income | 3000-5000 yuan | 1.711 | 0.582 | 8.650 | 5.537 | 1.770 | 17.322 | 0.003 |
|  |  | >5000 yuan | Ref |  |  |  |  |  |  |
|  | Degree of knowledge of the disease | Basic understanding | -2.272 | 1.100 | 4.269 | 0.103 | 0.012 | 0.890 | 0.039 |
|  |  | Partial understanding | Ref |  |  |  |  |  |  |
|  | Other chronic diseases | No | -1.788 | 0.780 | 5.256 | 0.167 | 0.036 | 0.771 | 0.022 |
|  |  | Yes | Ref |  |  |  |  |  |  |

Note: C1 = very low level rising group, C2 = low level rising group, C3 = medium level rising group, C4 = high level stable group.

Table S4. Univariate analysis of mental health trajectories in hysterectomy patients

| Variable | | C1（*n*=32） | C2（*n*=118） | C3（*n*=40） | *χ*^2^ | *P* |
| --- | --- | --- | --- | --- | --- | --- |
| Age | <40 | 3（9.4） | 2（1.7） | 0（0.0） | 6.484 | 0.126 |
|  | 40-59 | 24（75.0） | 99（83.9） | 31（77.5） |  |  |
|  | ≥60 | 5（15.6） | 17（14.4） | 9（22.5） |  |  |
| Marital status | Married | 26（81.3） | 96（81.4） | 37（92.5） | 2.884 | 0.236 |
|  | Single/Divorce/Widowed | 6（18.8） | 22（18.6） | 3（7.5） |  |  |
| Childbearing status | No | 4（12.5） | 11（9.3） | 5（12.5） | 0.745 | 0.664 |
|  | Yes | 28（87.5） | 107（90.7） | 35（87.5） |  |  |
| Education level | Elementary school and below | 2（6.3） | 21（17.8） | 5（12.5） | 10.994 | 0.084 |
|  | junior high school | 5（15.6） | 29（24.6） | 13（32.5） |  |  |
|  | High school or technical secondary school | 9（28.1） | 34（28.8） | 15（37.5） |  |  |
|  | College degree or above | 16（50.0） | 34（28.8） | 7（17.5） |  |  |
| Occupation status | Employed | 17（53.1） | 31（26.3） | 7（17.5） | 13.464 | 0.037 |
|  | Retired | 8（25.0） | 40（33.9） | 13（32.5） |  |  |
|  | Unemployed or jobless | 2（6.3） | 20（16.0） | 7（17.5） |  |  |
|  | Other | 5（15.6） | 27（22.9） | 13（32.5） |  |  |
| Average monthly household income | <3000 yuan | 2（6.3） | 22（18.6） | 8（20.0） | 13.009 | 0.011 |
|  | 3000-5000 yuan | 8（25.0） | 43（36.4） | 21（52.5） |  |  |
|  | >5000 yuan | 22（68.8） | 53（44.9） | 11（27.5） |  |  |
| Residence | City | 28（87.5） | 97（82.2） | 31（77.5） | 1.212 | 0.546 |
|  | Township or Rural | 4（12.5） | 21（17.8） | 9（22.5） |  |  |
| Payment methods | At own expense | 1（3.1） | 1（0.8） | 3（7.5） | 13.147 | 0.024 |
|  | Rural cooperative medical care | 1（3.1） | 10（8.5） | 2（5.0） |  |  |
|  | Resident medical insurance | 5（15.6） | 39（33.1） | 17（42.5） |  |  |
|  | Employee medical insurance | 25（78.1） | 68（57.6） | 18（45.0） |  |  |
| Degree of knowledge of the disease | Basic understanding | 30（93.8） | 89（75.4） | 25（62.5） | 9.484 | 0.008 |
|  | Partial understanding | 2（6.3） | 29（24.6） | 15（37.5） |  |  |
| Surgical indication | Benign | 29（90.6） | 104（88.1） | 16（40.0） | 44.290 | <0.001 |
|  | Malignant | 3（9.4） | 14（11.9） | 24（60.0） |  |  |
| Surgical approach | Laparoscopic surgery | 31（96.9） | 110（93.2） | 29（72.5） | 12.818 | 0.001 |
|  | Open surgery | 1（3.1） | 8（6.8） | 11（27.5） |  |  |
| Other chronic diseases | No | 27（84.4） | 92（78.0） | 25（62.5） | 5.440 | 0.066 |
|  | Yes | 5（15.6） | 26（22.0） | 15（37.5） |  |  |
| Time since diagnosis | <1 year | 12（37.5） | 49（41.5） | 18（45.0） | 2.869 | 0.580 |
|  | 1-5 years | 14（43.8） | 35（29.7） | 12（30.0） |  |  |
|  | >5years | 6（18.8） | 34（28.8） | 10（25.0） |  |  |
| Treatment methods | surgery | 31（96.9） | 112（94.9） | 22（55.0） | 35.354 | <0.001 |
|  | Surgery + Adjuvant Therapy | 1（3.1） | 6（5.1） | 18（45.0） |  |  |
| Length of hospital stay | ≤7 days | 12（37.5） | 34（28.8） | 5（12.5） | 39.786 | <0.001 |
|  | 8-14 days | 18（56.3） | 75（63.6） | 16（40.0） |  |  |
|  | >14 days | 2（6.3） | 9（7.6） | 19（47.5） |  |  |

Note: C1 = good mental health rising group, C2 = mental health risk reduction group, C3 = psychological problem relief group.

Table S5. Assigning values to independent variables of mental health trajectories

| Independent variable | Variable name | Assignment method |
| --- | --- | --- |
| Occupation status | X_1_ | Using “other” as the reference |
|  |  | Employed=1，Retired=0，Unemployed or jobless=0 |
|  |  | Employed=0，Retired=1，Unemployed or jobless=0 |
|  |  | Employed=0，Retired=0，Unemployed or jobless=1 |
| Payment methods | X_2_ | Using “Employee medical insurance” as the reference |
|  |  | At own expense =1，Rural cooperative medical care =0，Resident medical insurance =0 |
|  |  | At own expense =0，Rural cooperative medical care =1，Resident medical insurance =0 |
|  |  | At own expense =0，Rural cooperative medical care =0，Resident medical insurance =1 |
| Average monthly household income | X_3_ | Using “>5000 yuan” as the reference |
|  |  | <3000 yuan =1，3000-5000 yuan =0 |
|  |  | <3000 yuan =0，3000-5000 yuan =1 |
| Degree of knowledge of the disease | X_4_ | Basic understanding=1，Partial understanding=2 |
| Surgical indication | X_5_ | Benign=1，Malignant=2 |
| Surgical approach | X_6_ | Laparoscopic surgery=1，Open surgery=2 |
| Treatment methods | X_7_ | surgery=1，Surgery + Adjuvant Therapy=2 |
| Length of hospital stay | X_8_ | Using “>14days” as the reference |
|  |  | ≤7 days =1，8-14 days =0 |
|  |  | ≤7 days =0，8-14 days =1 |

Table S6. The logistic regression of mental health trajectories

| Group | Variable | | *B* | *SE* | *Waldχ*^2^ | *OR* | 95%*CI* | | *P* |
| --- | --- | --- | --- | --- | --- | --- | --- | --- | --- |
|  |  |  |  |  |  |  | Lower | Upper |  |
| C1 VS C3 | Surgical indication | Benign | 1.975 | 0.807 | 5.992 | 7.210 | 1.483 | 35.064 | 0.014 |
|  |  | Malignant | Ref |  |  |  |  |  |  |
|  | Degree of knowledge of the disease | Basic understanding | 2.280 | 0.848 | 7.224 | 9.780 | 1.854 | 51.590 | 0.007 |
|  |  | Partial understanding | Ref |  |  |  |  |  |  |
| C2 VS C3 | Surgical indication | Benign | 1.708 | 0.559 | 9.330 | 5.520 | 1.844 | 16.518 | 0.002 |
|  |  | Malignant | Ref |  |  |  |  |  |  |
|  | Treatment methods | surgery | 1.450 | 0.666 | 4.734 | 4.261 | 1.155 | 15.726 | 0.030 |
|  |  | Surgery + Adjuvant Therapy | Ref |  |  |  |  |  |  |

Note: C1 = good mental health rising group, C2 = mental health risk reduction group, C3 = psychological problem relief group.

Table S7. Conditional and joint probabilities of post-traumatic growth and mental health trajectory groups with 95% confidence intervals

| PTG Trajectory | MH Trajectory | *P* (MH \| PTG) % (95% CI) | *P* (PTG \| MH) % (95% CI) | Joint probability % (95% CI) |
| --- | --- | --- | --- | --- |
| 1 | 1 | 8.8 (0.0–34.4) | 8.0 (0.0–20.9) | 1.3 (0.0–4.2) |
|  | 2 | 41.7 (25.4–81.4) | 10.1 (18.8–56.8) | 6.3 (2.1–11.1) |
|  | 3 | 49.5 (8.3–64.3) | 36.5 (0.0–46.7) | 7.5 (0.5–10.5) |
| 2 | 1 | 13.4 (3.0–39.5) | 28.4 (3.2–18.2) | 4.8 (1.1–15.8) |
|  | 2 | 63.3 (40.2–78.1) | 36.0 (22.2–65.2) | 22.6 (11.3–36.8) |
|  | 3 | 23.3 (5.1–42.5) | 40.3 (9.3–55.2) | 8.3 (2.1–14.5) |
| 3 | 1 | 10.2 (3.3–41.3) | 21.0 (2.3–56.6) | 3.5 (1.1–14.0) |
|  | 2 | 77.7 (48.8–86.3) | 42.7 (9.1–53.2) | 26.7 (12.6–39.0) |
|  | 3 | 12.1 (0.0–25.6) | 20.2 (2.6–21.5) | 4.2 (0.0–10.5) |
| 4 | 1 | 48.4 (11.7–74.5) | 42.6 (10.0–51.1) | 7.1 (1.1–11.1) |
|  | 2 | 47.6 (22.0–82.0) | 11.2 (23.2–60.5) | 7.0 (1.6–14.2) |
|  | 3 | 4.0 (0.0–18.0) | 3.0 (0.0–16.7) | 0.7 (0.0–3.2) |

Note: PTG Trajectory: 1=very low level rising group, 2=low level rising group, 3 = medium level rising group, 4=high level stable group. MH Trajectory: 1 = good mental health rising group, 2 = mental health risk reduction group, 3 = psychological problem relief group. *P* (MH | PTG), conditional probability of mental health trajectory given post-traumatic growth trajectory; *P* (PTG | MH), conditional probability of post-traumatic growth trajectory given mental health trajectory.

Point estimates are from the original model; 95% CIs are from 1,000 bootstrap resamples. For two small subgroups (PTG=1/MH=2; PTG=4/MH=2), the bootstrap distribution shifted, so the point estimates fall outside the CIs. These two estimates should be interpreted with caution.
